# Comparison of Neural Responses to Cat Meows and Human Vowels in the Anterior and Posterior Auditory Field of Awake Cats

**DOI:** 10.1371/journal.pone.0052942

**Published:** 2013-01-02

**Authors:** Hanlu Ma, Ling Qin, Chao Dong, Renjia Zhong, Yu Sato

**Affiliations:** 1 Department of Physiology, Interdisciplinary Graduate School of Medicine and Engineering, University of Yamanashi, Chuo, Yamanashi, Japan; 2 Department of Physiology, China Medical University, Shenyang, People’s Republic of China; Tokyo Medical and Dental University, Japan

## Abstract

For humans and animals, the ability to discriminate speech and conspecific vocalizations is an important physiological assignment of the auditory system. To reveal the underlying neural mechanism, many electrophysiological studies have investigated the neural responses of the auditory cortex to conspecific vocalizations in monkeys. The data suggest that vocalizations may be hierarchically processed along an anterior/ventral stream from the primary auditory cortex (A1) to the ventral prefrontal cortex. To date, the organization of vocalization processing has not been well investigated in the auditory cortex of other mammals. In this study, we examined the spike activities of single neurons in two early auditory cortical regions with different anteroposterior locations: anterior auditory field (AAF) and posterior auditory field (PAF) in awake cats, as the animals were passively listening to forward and backward conspecific calls (meows) and human vowels. We found that the neural response patterns in PAF were more complex and had longer latency than those in AAF. The selectivity for different vocalizations based on the mean firing rate was low in both AAF and PAF, and not significantly different between them; however, more vocalization information was transmitted when the temporal response profiles were considered, and the maximum transmitted information by PAF neurons was higher than that by AAF neurons. Discrimination accuracy based on the activities of an ensemble of PAF neurons was also better than that of AAF neurons. Our results suggest that AAF and PAF are similar with regard to which vocalizations they represent but differ in the way they represent these vocalizations, and there may be a complex processing stream between them.

## Introduction

One of the important physiological roles of the auditory system is to discriminate the communication sounds generated by conspecies. To reveal the underlying neural mechanism, many electrophysiological studies have investigated the neural activities of the auditory cortex in response to conspecific vocalizations. Currently, the accumulated data support that the acoustic features of vocalizations are represented by the primary auditory cortex (A1) of various species in a spatially distributed fashion [1]–[8]. In other words, a single A1 neuron encodes simple acoustic features such as the frequency component or temporal envelope, and a population of neurons is elicited by a vocalization in a specific temporal and spatial sequence, which presents a population coding of vocalizations.

As for how the vocalizations are processed in the auditory cortices outside A1, no common conclusion has been reached. In the visual system, information being transferred from the primary visual cortex to extrastriated visual cortex constitutes two processing streams: a ventral or ‘what’ processing stream and a dorsal or ‘where’ processing stream, which is involved in the object vision and spatial vision, respectively [9]–[11]. In parallel with the visual system, Rauschecker and colleagues proposed a concept of two streams of auditory cortical processing: a posterior/dorsal stream dealing with the processing of spatial aspects of sound (“where” stream), and an anterior/ventral stream for the identification of sounds such as species-specific vocalizations (“what” stream) [12]–[15]. The “what” stream originates in A1 and includes a series of projections through the antero-lateral belt auditory cortex (AL), the dorsal bank of the superior temporal sulcus (STS), and ultimately to the ventro-lateral prefrontal cortex (vPFC). Inspired by this concept, various studies have been conducted on monkeys to investigate conspecific vocalization-evoked responses in AL, STS and vPFC [16]–[21]. Neurons in these regions generally show a preference for monkey calls over other complex and simple sounds. Although there is some controversy about call selectivity in the neurons of different regions [16]–[20], these studies suggest the existence of a vocalization-processing hierarchy in the non-human primate cortex.

In contrast to the extensive studies of monkeys, electrophysiological investigations into the vocalization-processing hierarchy have rarely been conducted using other mammals. Cats are also a well-used model in auditory neuroscience research, because cat audibility is broad, highly overlapping that of humans, and because the majority of auditory areas are easily approachable, as they are exposed on the surfaces of gyri, rather than being buried in the depths of a sulcus. The auditory cortex of the cat has been divided into at least 13 distinct fields on the basis of anatomy, physiology, and behavior [22]–[26]. Among these, A1, the anterior auditory field (AAF) and posterior auditory field (PAF) are three neighboring regions having a tonotopic map. AAF lies just rostral to A1; the anterior ectosylvian sulcus (AES) is the anatomic boundary between A1 and AAF. PAF lies caudal and ventral to A1; the posterior ectosylvian sulcus (PES) is the anatomic boundary between them. Reversals of frequency representation appear near the AES and PES, further delineating the three regions. As mentioned above, the neuronal responses to conspecific vocalizations have been well analyzed in A1 of cats [2], [6], [7], whereas few data from AAF and PAF are available. Recently, a behavioral experiment showed that bilateral deactivation of the AAF resulted in deficits in a pattern-discrimination task (discriminating different gap sequences embedded in broadband noise bursts), whereas bilateral deactivation of the PAF resulted in deficits in a sound-localization task [27]. This result suggests that AAF may be more involved in sound identification than PAF; however, this possibility needs to be verified by comparing the electrophysiological results between the two cortical fields. Two previous studies recorded the neural responses to conspecific vocalizations in anesthetized cats in several cortical areas, including AAF and PAF [2], [28], and found little discrimination of cat vocalizations in AAF and PAF and only reported response latency differences. To further clarify the roles of AAF and PAF in vocalization processing, in the present study, we recorded single-unit activities in the AAF and PAF of awake cats as they passively listened to five conspecific vocalization exemplars presented in forward and time-reversed directions and five human vowels. Time-reversed meows have all the acoustic features of natural meows, with the exception that the temporal order is reversed. Human vowels share many similar acoustic features with meows, such as slow temporal dynamics, low spectral range, and a harmonic stack structure. We found that the neural responses to these slowly-changing, harmonic sounds in PAF were more complex and had longer latency than those in AAF. Our results suggest that AAF and PAF are similar with regard to which vocalizations they represent but differ in the way they represent these vocalizations. The stream of vocalization processing may not completely separate at AAF and PAF, and a more complex scheme of vocalization processing may activate in the early auditory cortical field of cats.

## Results

**Figure 1:**
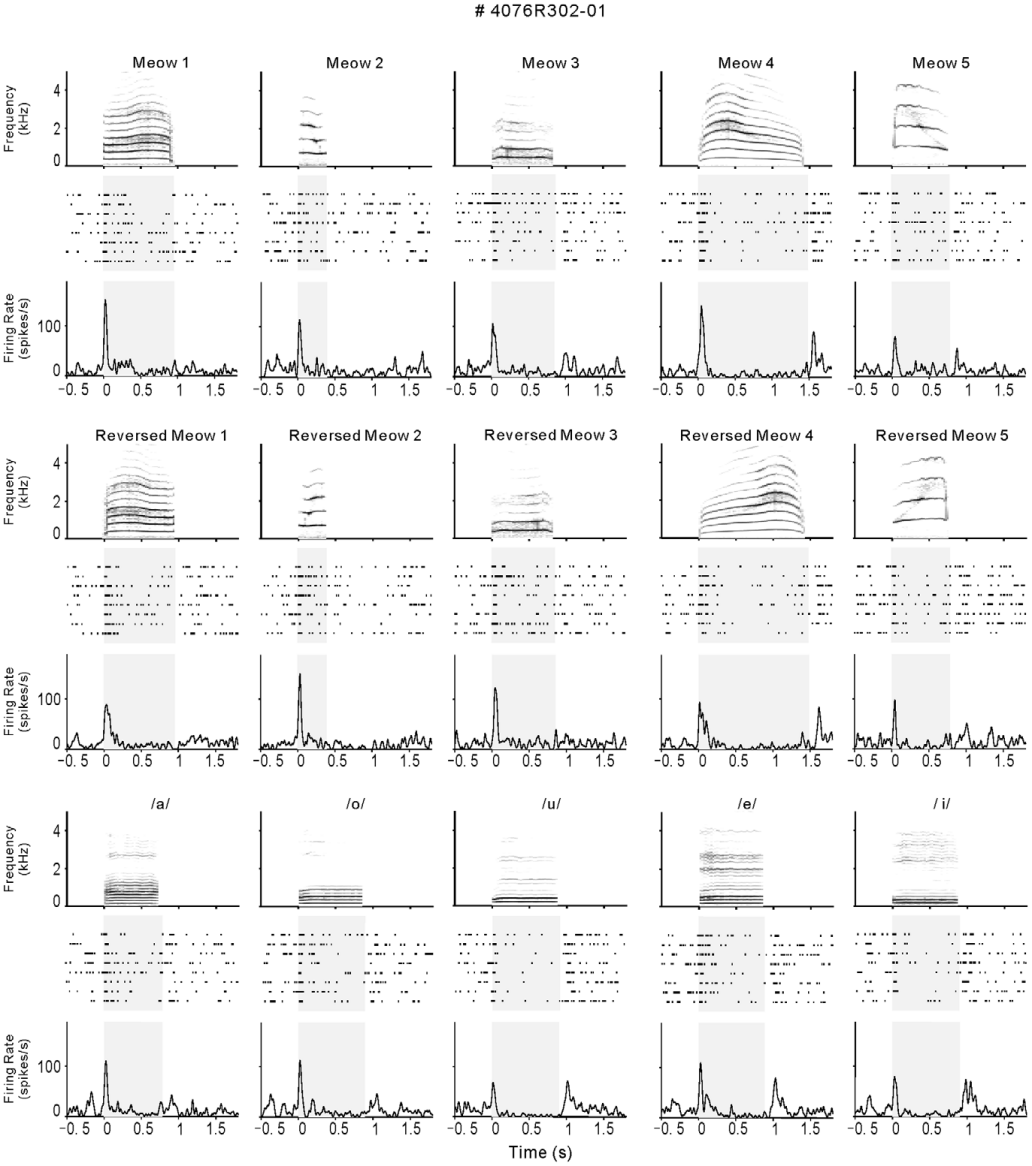
Responses to the fifteen different stimuli by a representative AAF neuron. In each panel, the top shows the sonogram of the stimulus with the y-axis denoting the frequency from 0 Hz (bottom) to 5 kHz (top) on a linear scale. Time is on the x-axis, and the gray scale displays the energy of the stimulus, with darker colors indicating greater power. Below the sonograms are the spike rasters. Each line is a different trial (bottom line is the first trial of that stimulus type) and each dot mark represents a single spike. Below this is the post-stimulus time histogram (PSTH). Bin size is 1 ms, smoothed by with a Gaussian kernel (σ = 10). A–E show forward meows, F–H are time-reversed meows, K–O are human vowels. This neuron shows similar temporal patterns of response to all the stimuli.

**Figure 2:**
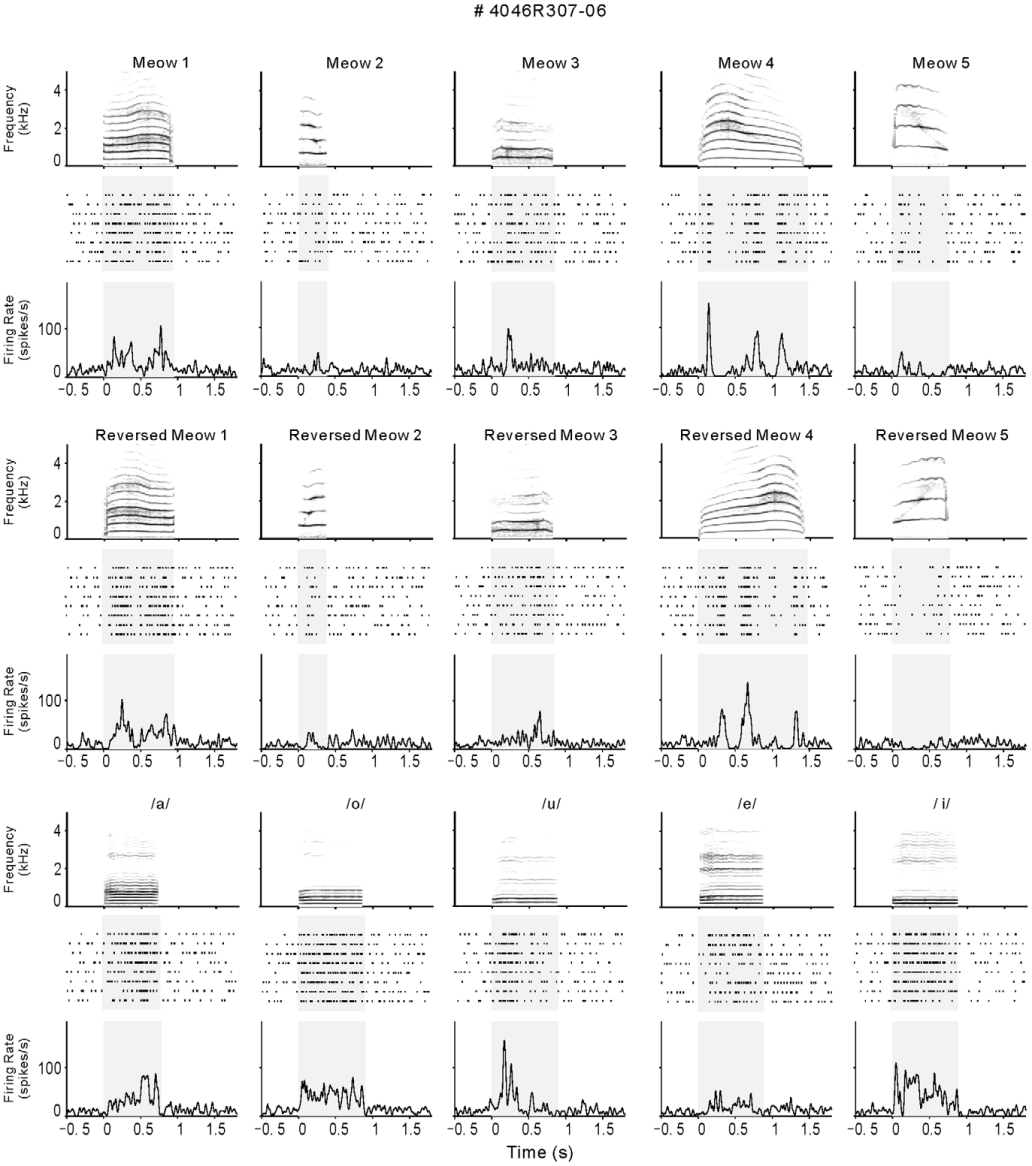
Responses by an example cell of PAF. Conventions as in Fig. 1. This neuron shows various temporal patterns of response to the stimuli.

We conducted extracellular single-unit recording in both hemispheres of 2 awake cats, and collected spike activities of 194 well-isolated single units that showed a significant response to at least one of the 15 stimuli tested. Single-unit activities were recorded at a depth of 400–2,000 µm from the first encountered unit of each track. According to the histological reconstruction of recording sites, 92 units were identified in AAF and the remaining 102 units were in PAF. The cortical neurons showed different firing patterns in response to the vocalization stimuli. Two examples of the neural responses are presented in Figs. 1 and 2.

### Representative Examples of Neural Responses to Vocalizations

A response profile from one AAF neurons is shown in Fig. 1. The top panel shows the sonogram of the stimulus, with frequencies from 0 to 5 kHz along the y-axis (bottom to top) with the darker colors showing increasing amounts of power. Below each sonogram is the dot raster, showing the response of the neuron to each presentation of that stimulus. Each dot represents a single action potential and each row shows a single trial. The PSTH is shown below the rasters. The neuron in Fig. 1 is typical of the sample in AAF that showed a transient excitatory response at the onset of all the stimuli. An excitatory response was also observed at the offset of some stimuli (i.e./u/and/e/). During the stimulus period, the neuron’s firing rate was significantly suppressed by some stimuli, which was designated as a suppressive response (see Materials and Methods).

The example neuron of PAF in Fig. 2 showed more variability of response pattern. The excitatory response of this neuron continued throughout the stimulus period in some stimuli (/o/and/i/), while it transiently occurred in others (Meow 3 and/u/). More specifically, the responses to forward and reversed Meow 4 showed three excitatory peaks, which were separated by suppressive valleys. This neuron showed a continuous suppressive response to reversed Meow 5. The various response patterns may contain more information to identify different vocalizations.

### Population Neural Responses to Vocalizations

**Figure 3:**
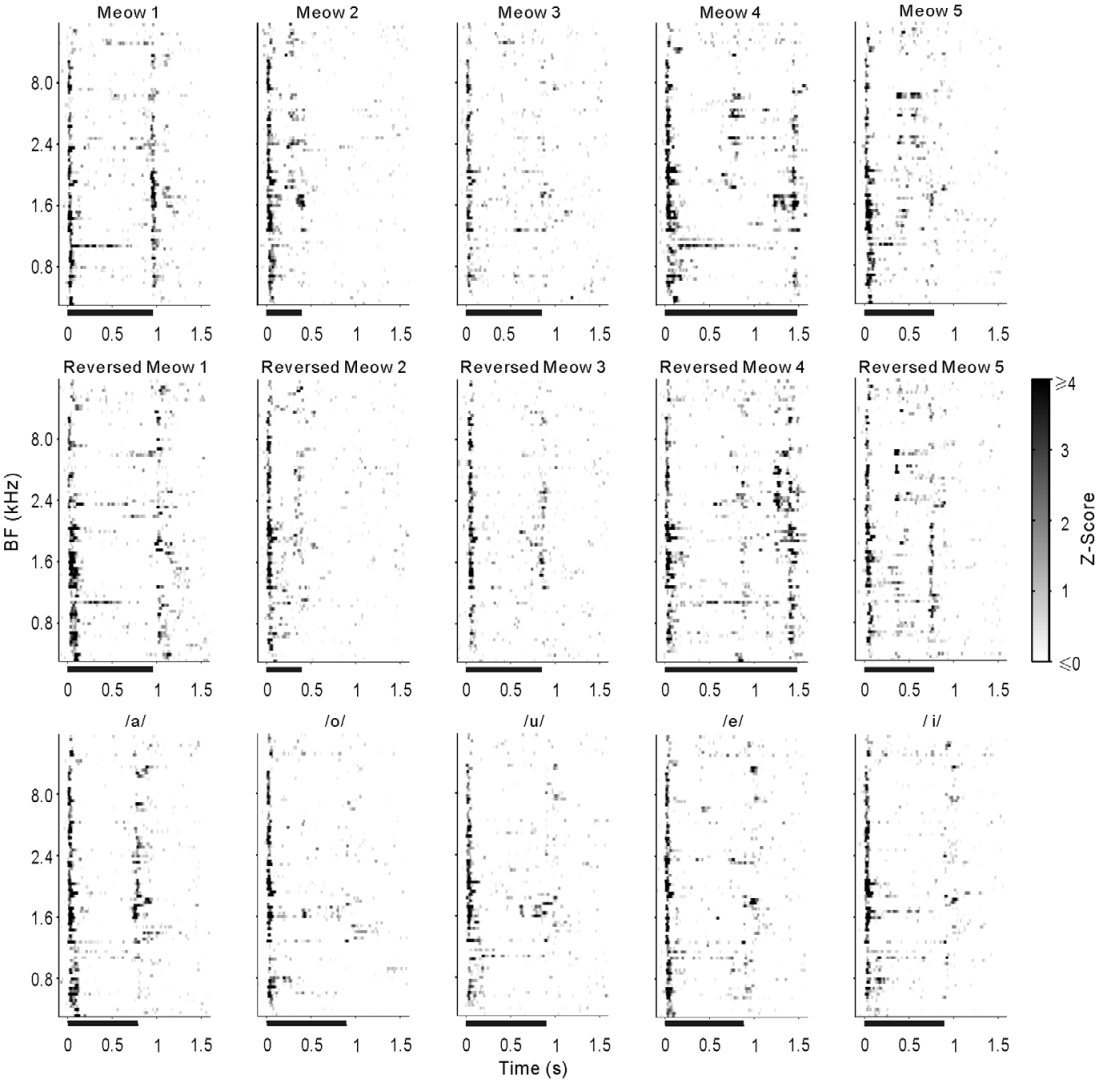
Individual PSTHs depicting the response patterns of 92 AAF neurons to different stimuli. The absolute firing rates of each neuron were normalized to Z-scores and, for visualization purposes, smoothed and displayed in grayscale plots. PSTHs are aligned at the onset of tone. Black horizontal bars at bottom of plot show the duration of stimulus. Within each plot, neurons were ranked by the neuron’s BF.

**Figure 4:**
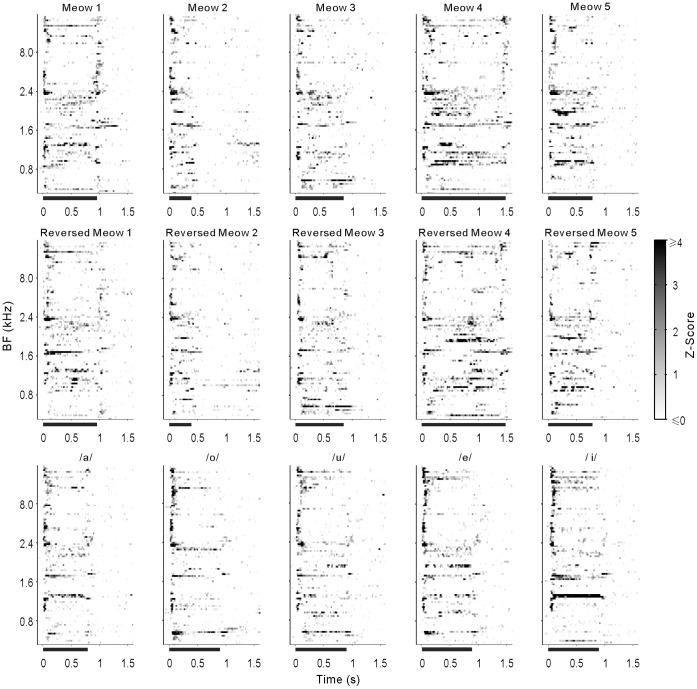
Individual PSTHs of 102 PAF neurons. Conventions as in Fig. 3.

**Figure 5:**
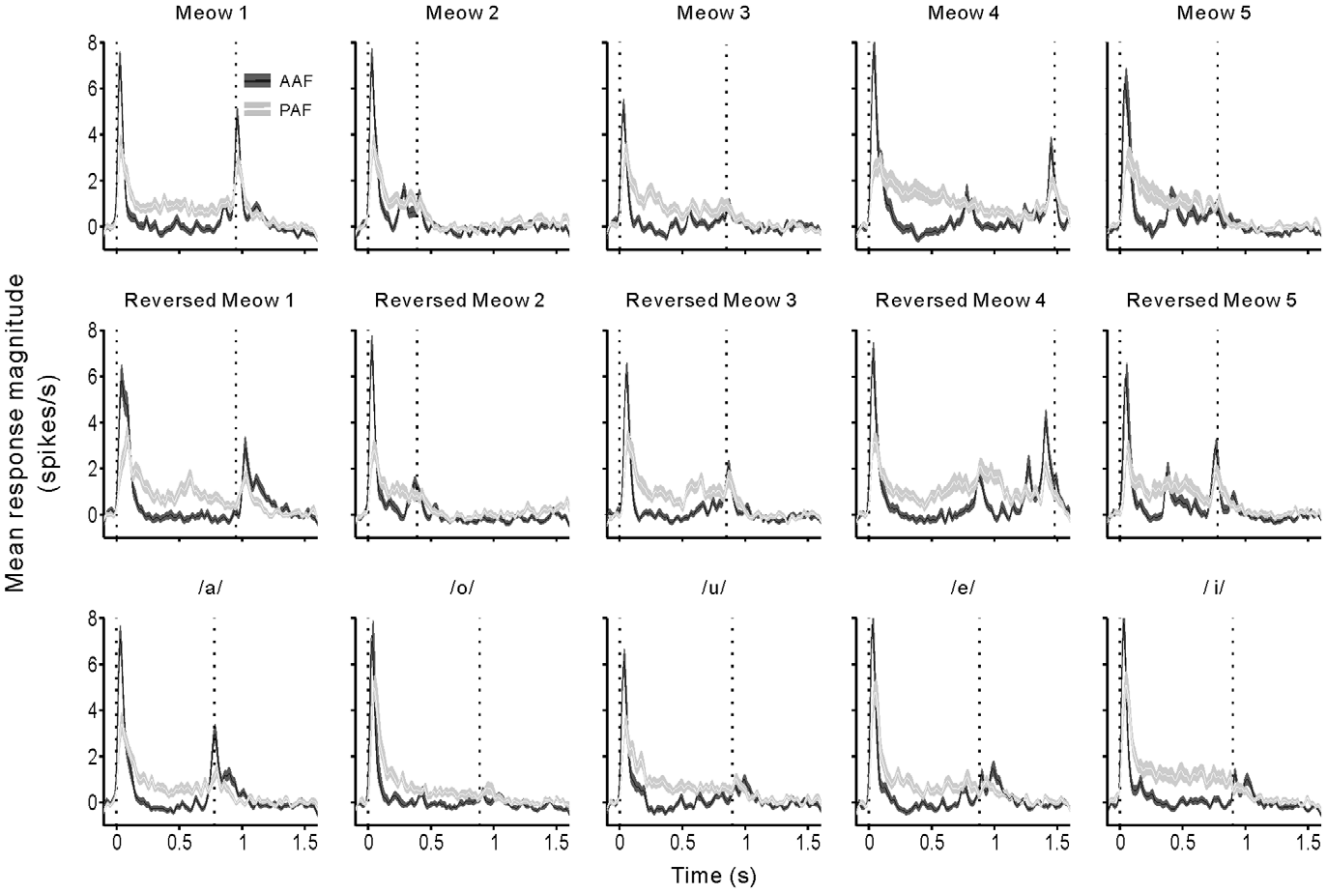
Comparison of the mean PSTHs averaged across all AAF and PAF neurons. Black and white curves represent the mean PSTHs of AAF and PAF, respectively. Shaded area represents SE. Vertical dashed lines indicate the onset and offset of stimulus, respectively.

To illustrate the difference between the response patterns of AAF and PAF neurons, we constructed the Z-score PSTH of all the recorded neurons (see Materials and Methods). Figures 3 and 4 show a stack of Z-score PSTHs of the 15 stimuli for 92 AAF and 102 PAF neurons, respectively. In each panel, the PSTHs from different neurons are arranged in ascending order of the best frequency (BF), which was the frequency of pure tone that evoked the largest excitatory response of the neurons. Because we only selected neurons that showed a significant response to at least one of the vocalization stimuli in this study, the distribution of BF was obviously biased to the low frequency side in both AAF and PAF populations. Accordingly the ordinate of Figs. 3 and 4 is plotted on a non-linear scale. The 25%, 50% and 75% percentiles of BF distribution were 1.5, 2.0 and 4.3 kHz in AAF and 0.7, 2.4 and 4.6 Hz in PAF, respectively. There was no significant difference between the BF distributions of AAF and PAF neuron samples (p = 0.7, Mann-Whitney *U*-test). As represented by the example in Fig. 1, the majority of AAF neurons showed a transient excitatory response at the onset and/or offset of vocalizations (Fig. 3). In contrast, PAF neurons showed less pronounced responses at the onset and offset of stimulus, and more PAF neurons, especially those with BF between 0.8 and 4.5 kHz, showed a sustained excitatory response during the stimulus period (Fig. 4). We then compared the average PSTHs across all neurons of AAF and PAF in Fig. 5. It is clear that AAF showed a sharper and higher onset/offset response, while PAF showed a stronger sustained response during the stimulus period; also, the latency of response peak was longer in PAF than in AAF.

### Quantitative Comparison of Neural Response Properties between AAF and PAF

**Figure 6:**
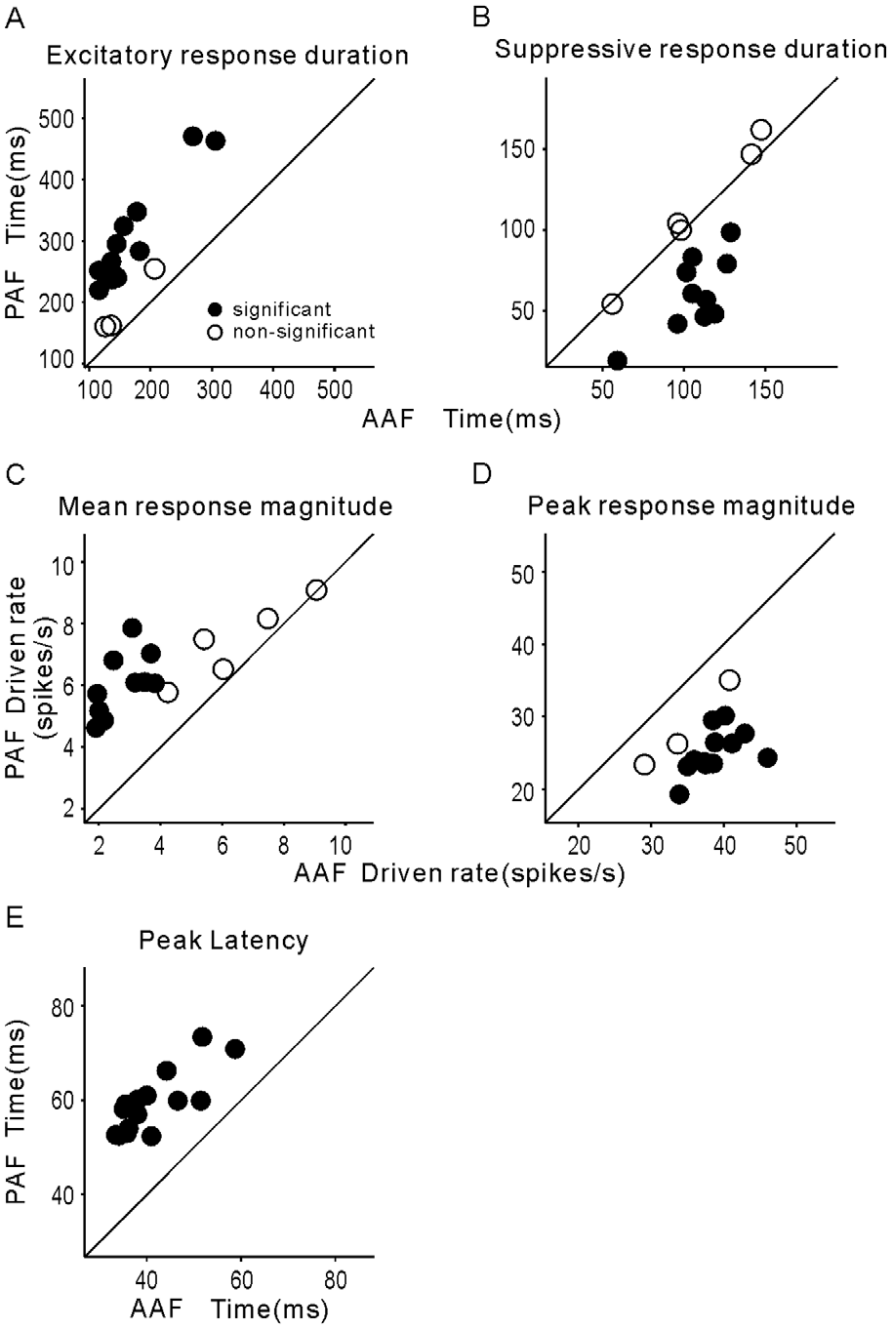
Comparison of response properties of AAF and PAF neurons. A: mean of excitatory response duration averaged over 102 PAF neurons against those of 92 AAF neurons. Each symbol represents the mean value of one stimulus. Filled symbol indicates that the difference of mean values between AAF and PAF neurons is statistically significant (p<0.05, t-test). Open symbol indicates that the difference is non-significant. B: comparison of suppressive response durations. C: comparison of mean response magnitudes. D: comparison of peak response magnitudes. E: comparison of peak latencies.

We further used several parameters to quantify the observed differences between the neural response properties of AAF and PAF. Firstly, we measured the response duration of each neuron evoked by each stimulus (see Materials and Methods). The mean duration of excitatory response averaged across the 102 PAF neurons was plotted against that of 92 AAF neurons in Fig. 6A (each symbol represents the result of one stimulus). For all 15 stimuli, the mean response duration of PAF neurons was longer than that of AAF neurons. The difference in the excitatory response duration was statistically significant in 12 of the 15 stimuli (t-test, p<0.05, represented by filled circles in Fig. 6A). The mean duration of the suppressive response was significantly shorter for PAF versus AAF neurons for 10/15 stimuli (Fig. 6B).

Because the suppressive response was defined as a decrease of the firing rate from the spontaneous level, the observed difference may be attributable to a difference in the spontaneous firing rates of AAF and PAF neurons; however, this was not true in our dataset, in which the mean and SD of spontaneous firing rates were 10.4±6.1 and 8.7±6.2 spikes/s in AAF and PAF, respectively. No significant difference was found between them (p>0.1, t-test).

Secondly, we compared the response magnitudes between AAF and PAF. The mean response magnitude was defined as the mean firing rate during the entire stimulus duration and 50 ms post-stimulus duration. As illustrated in Fig. 6C, the mean response magnitudes in PAF neurons were significantly higher than those in AAF neurons for 10 stimuli (p<0.05, t-test). When comparing just the peak response magnitude around the 50 ms period when the PSTH reached its maximum, AAF neurons generally showed a stronger maximum response than PAF neurons (Fig. 6D). The difference was statistically significant (p<0.05, t-test) in 12 stimuli, indicating that the transient responses of AAF neurons were more pronounced than the extended responses of PAF neurons.

Thirdly, we examined the latency of the peak response (peak latency) to each stimulus. The mean latency of PAF was significantly longer (p<0.05, t-test) than that of AAF in all 15 stimuli (Fig. 6E). This result is consistent with a recent report on anesthetized cats in which the response latency was faster in AAF than in PAF [28].

**Figure 7:**
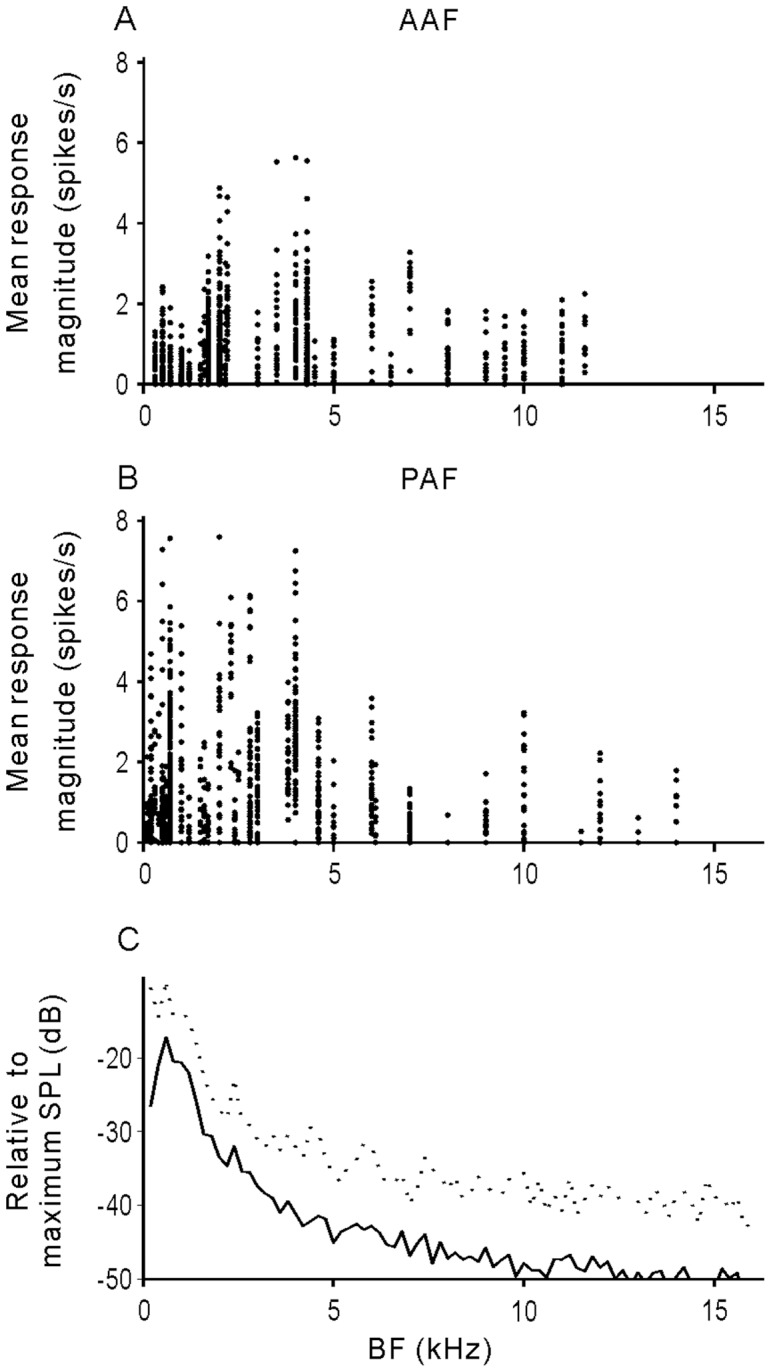
Relation between response magnitude and BF. A and B: mean response magnitude of all 15 vocalizations versus the BF of AAF and PAF neurons, respectively. C: Energy distribution of our vocalization stimuli. Solid line indicates the mean SPL of the 15 vocalizations. Dotted line indicates the mean+SD. Each spectrum of vocalization was normalized by its maximum as 0, before calculating the mean and SD.

Previous studies have shown that the neural response was dependent on the relation of the sound spectrum and the neuron’s pure-tone tuning property [29]–[32]. This was also examined by plotting the magnitude of the vocalization-evoked response against BF. Figure 7A and 7B show the scatter plot of the mean response magnitude for all 15 stimuli versus neuron BF in AAF and PAF. For comparison, the mean spectrum averaged across the 15 stimuli is plotted in Fig. 7C (individual spectra were normalized by their maximum SPL as 0 before averaging). It is apparent that higher response magnitudes were more frequently found in the BF region below 5 kHz, where the stimulus energy is located. During the experiments, we also recorded 54 neurons (23 neurons in AAF, 31 neurons in PAF) that responded to pure tones but not to vocalizations. The measurable BF of these neurons ranged from 10.8 kHz to 32 kHz (mean ± SD: 17.2±4.2 kHz) in AAF, and from 9.4 kHz to 30.5 kHz (mean ± SD: 15.4±5.6 kHz) in PAF, which were far from the region of high power in the vocalizations. Therefore, the energy distribution of vocalizations can be approximately reflected by the response magnitude distribution along the BF axis in both AAF and PAF.

### Neural Selectivity for Vocalization Sounds

**Figure 8:**
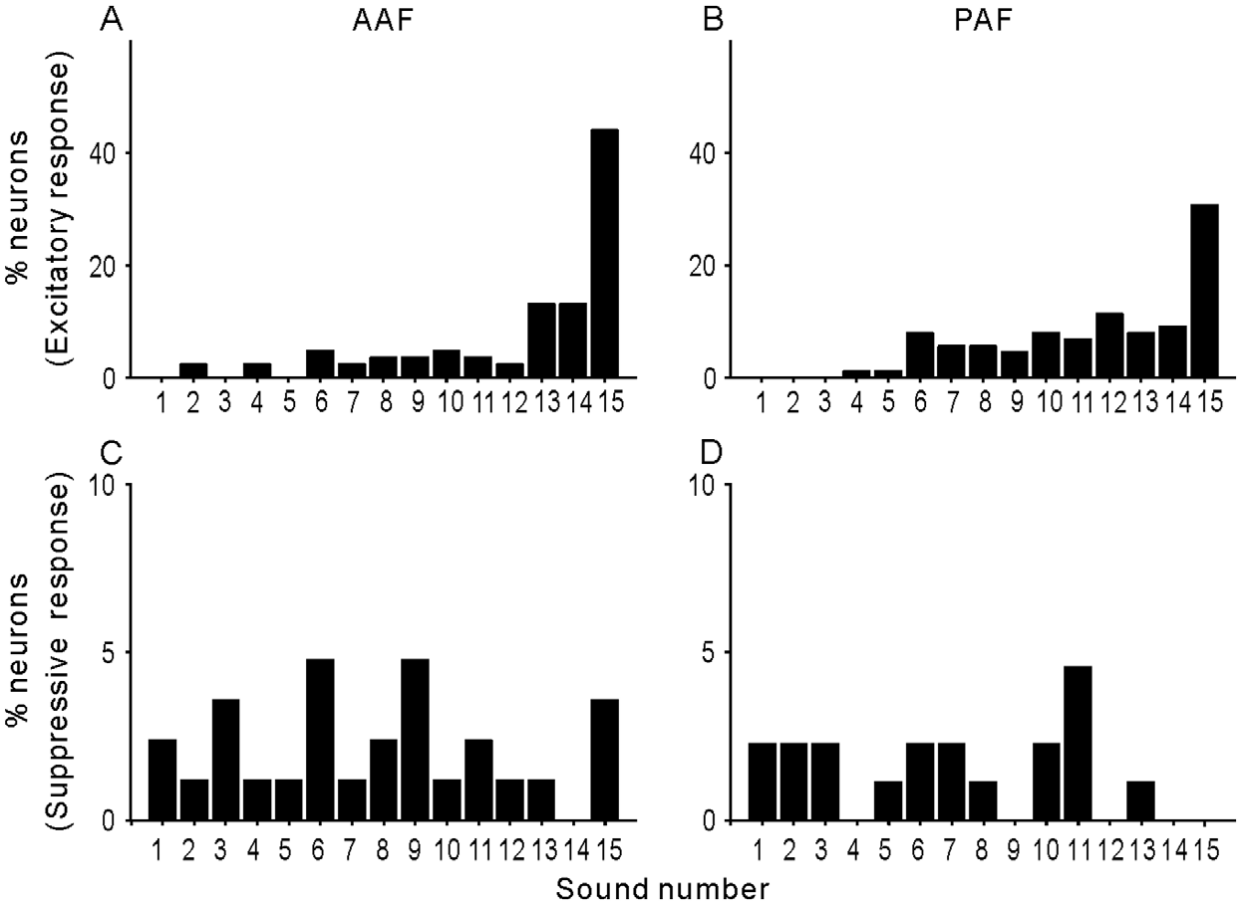
Neural selectivity of vocalization stimuli. A and B: percentage distribution of Nef among 92 AAF and 102 PAF neurons. Nef was calculated as the number of stimuli evoking an excitatory response in each neuron. C and D: percentage distribution of the Nef of suppressive response.

We next evaluated the neuron’s selectivity between different vocalizations by calculating the number of vocalizations in our dataset that elicited a significant excitatory response for each neuron [21], such that neurons that responded to many of the stimuli in the stimulus set were not selected. Figure 8A and B show the distribution of the number of sound elicited excitatory responses in the AAF and PAF neurons, respectively. In AAF (Fig. 8A), the majority of AAF neurons had poor selectivity for vocalizations. The selectivity increased in PAF (Fig. 8B), where fewer neurons responded to all stimuli in our stimulus set. According to this simple index of selectivity, AAF and PAF were not different (p = 0.47, Mann-Whitney U-Test).

The same analysis as described above for excitatory responses was performed for suppressive responses. Overall, 67.9% AAF neurons and 78.4% PAF neurons had no significant suppressive response to any stimuli. Of the remaining neurons, the number of sound-elicited suppressive response was evenly distributed from 1 to 15 (Fig. 8C and D) in both AAF and PAF. No significant difference was found between them.

### Neuronal Responses to Stimuli in Different Categories

**Figure 9:**
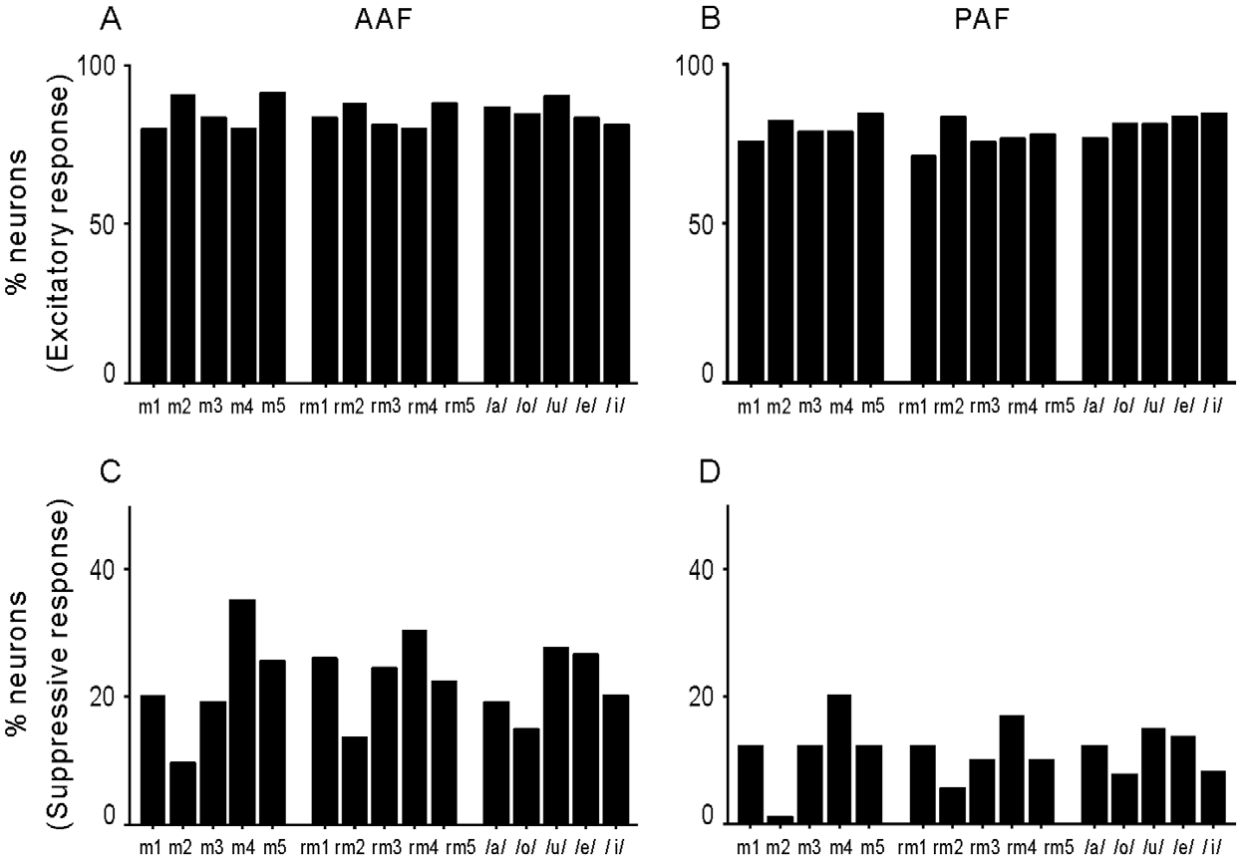
Effectiveness of each stimulus to evoke a neural response. A and B: percentage of neurons showing an excitatory response to each stimulus in AAF and PAF. C and D: percentage of neurons showing a suppressive response to each stimulus in AAF and PAF.

The next consideration was whether stimuli from 3 different categories (forward, reversed meows and human vowels) activated AAF and PAF neurons with different efficacy. Prior studies have reported a strong stimulus preference for forward versus reversed animal vocalizations. Since our dataset includes both forward and reversed vocalizations, a bias for forward sounds would be associated with a high number of neurons responding significantly to a subset of sounds in the stimulus set (e.g. forward meows 1–5). The percentage of neurons with significant responses was evenly distributed across all sounds in the stimulus set (Fig. 9A and B). Two-way ANOVA showed that the main effects of the stimulus category and cortical area and their interaction were not statistically significant (p>0.05). This indicates minimal selectivity for any sounds in the set, including forward versus reverse oriented “meows”. This may be attributed in part to the fact that cat vocalizations (and vowels) have very similar frequency and harmonic composition when played in forward and reversed directions.

On the other hand, the percentage of neurons with a suppressive response was higher in AAF neurons than in PAF neurons (Fig. 9C and D). Two-way ANOVA showed that there was a significant main effect of cortical area (p<0.01), but not of stimulus category or their interaction (p>0.05). This confirmed the above result (Fig. 6B) that AAF neurons were more suppressed by the stimuli. Although no significant difference in suppressive response was found among different stimulus categories, the forward and reversed Meow 4 evoked a larger percentage of suppressive responses, and the forward and reversed Meow 2 evoked a smaller percentage of suppressive responses in both AAF and PAF neurons. Because Meow 4 and Meow 2 were the longest and shortest meows in our exemplars, respectively, this result suggests that the suppressive response was more frequently found in a long stimulus.

We then compared the mean response magnitudes evoked by stimuli in different categories (forward, reversed meows and human vowels). In both AAF and PAF, no significant difference of mean response magnitude was found among the three stimulus categories (p>0.05, ANOVA, data not shown). Also, the peak response magnitudes were similar among different stimulus categories (p>0.05, ANOVA).

### Information Theory Analyses

**Figure 10:**
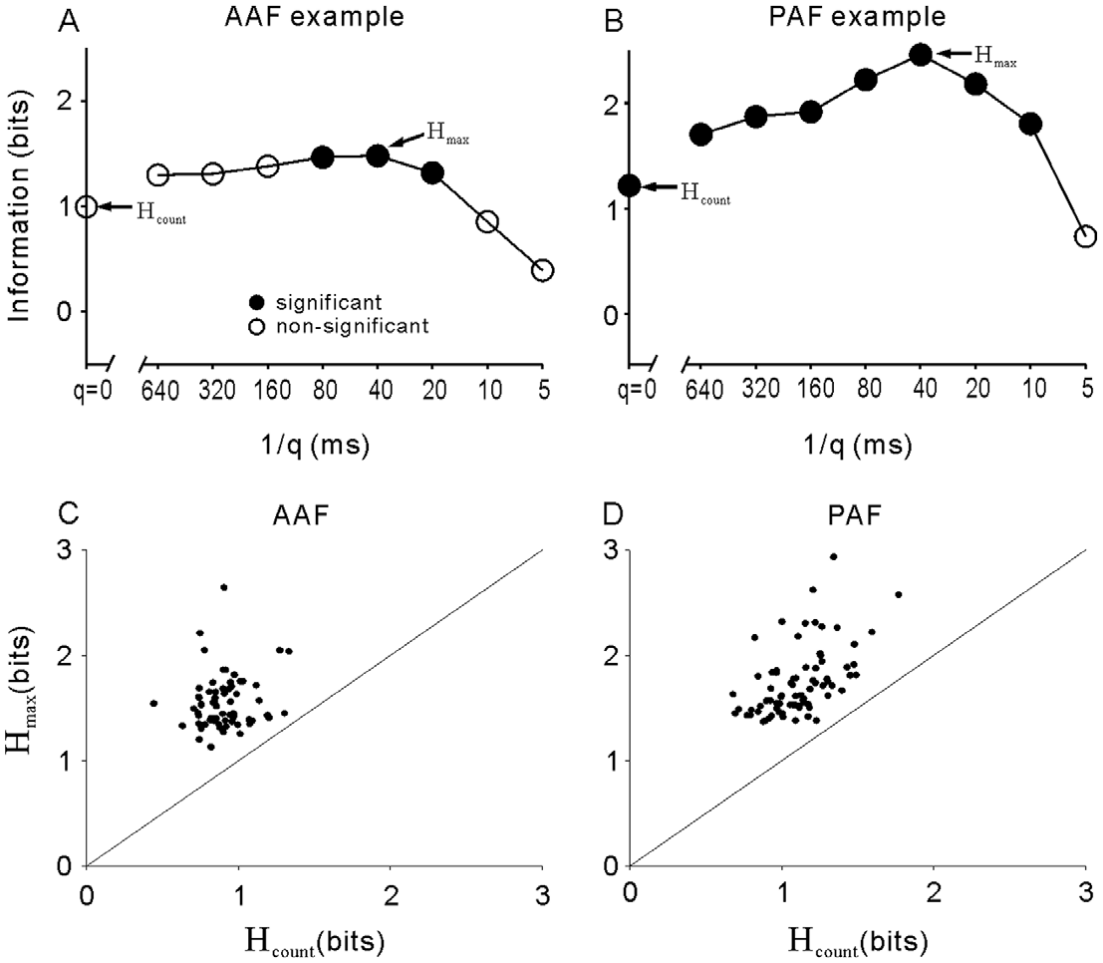
Stimulus information transmitted by AAF and PAF neurons. A and B: transmitted information as a function of the temporal precision (1/q) of the example AAF and PAF neurons shown in Figs 1 and 2. Filled symbols represent the value of information is significantly higher than the chance level (H_shuffled_ +2SD). C and D: scatters of the highest value of information (H_max_) versus the information obtained when only the firing rate is considered (H_count_ at q = 0) in AAF and PAF neurons. Diagonal line corresponds to H_count_ = H_max_.

The above analyses based on the mean response magnitude indicated that most neurons had low selectivity for different vocalizations; however, the variety of temporal response patterns may carry some decoding information for different stimuli. To confirm this possibility, we applied the metric-space method to estimate the amount of information about stimulus identity (H), carried by spike count and the precise timing of the spikes (see Materials and Methods for details). For each recorded neuron, we independently calculated the amount of information carried by the spike trains within 0–1.56 s after stimulus onset as a function of the temporal precision (1/q) relevant to spike timing. Because the durations of vocalization stimuli were different, we adopted the longest analysis time window to include all the stimulus durations, considering that the difference between spike activities during the later period of long stimuli and the post-stimulus activities of short stimuli also contributes to stimulus discrimination. The function of information against the temporal precision of the two example neurons in Figs. 1 and 2 is presented in Fig. 10A and B, respectively. The functions have been corrected by subtracting the mean H_shuffled_, obtained from randomly shuffled trials, from the H obtained from original data. Black dots represent that H was significant higher than the level of chance (>H_shuffled_ +2SD). The abscissa represents the temporal precision q ranging from 0 to 5 ms^−1^ in logarithmic steps. When q = 0, H only depends on the spike count (H_count_), and as q increases (1/q decreases), the analysis becomes increasingly sensitive to finer temporal features (spike time coding). For the example AAF neuron (Fig. 10A), H was non-significant at q = 0, but significant when 1/q was between 20 and 80 ms, indicating that the mean firing rate of this neuron did not transmit the stimulus information, but the temporal response patterns in 20–80 ms precision carried some decoding information. The example PAF neuron (Fig. 10B) was informative when just evaluating the mean firing rate (H_count_ was significant), and the amount of information increased when the spike trains were analyzed with better temporal precision. On such curves of H, the point at which significant H reaches its highest value (H_max_) indicates the extent to which correct discrimination between stimuli can be achieved by looking at the spike trains.

In our dataset, although all neurons were responsive to vocalizations (showed a significant response to at least one of the 15 vocalizations), they were not all informative for vocalization coding. The percentage of neurons that showed a significant H at one or more temporal precisions was 72% and 75% in AAF and PAF, respectively. For all these neurons, the value of H_max_ was plotted against the value of H_count_ in Fig. 10C and D. In both AAF and PAF, all the dots are located above the diagonal line (H_max_>H_count_), which means that taking the temporal precision into account increased the amount of transmitted information. Furthermore, the mean H_max_ of PAF neurons was significantly higher than that of AAF neurons (1.74±0.04 bit vs. 1.55±0.03 bit, p<0.001, t-test), indicating that the temporal responses of PAF neurons carried more stimulus information than those of AAF neurons.

### Linear Pattern Discriminator Performance

**Figure 11:**
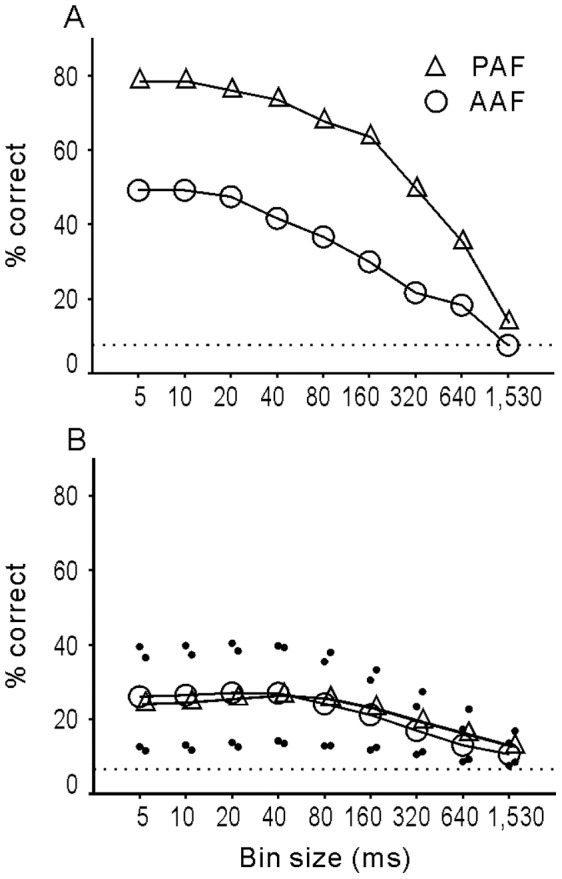
Accuracy of vocalization discrimination based on the spike activities of single neurons in AAF and PAF. A: percentage of correctly discriminated vocalizations as calculated from the example neurons in Fig. 1 (circles) and 2 (triangles), respectively. The percentage of correct classification is plotted as a function of the bin size. Dotted line indicates the chance performance of discrimination (6.7%). B: percentage of correct classification for the population of AAF and PAF neurons as a function of the bin size. Circles (AAF) and triangles (PAF) are the mean values and the dots represent the SD.

To further determine the extent to which neurons in different areas could use temporal pattern information in discriminating between vocalizations, a linear pattern discriminator model was applied based on the Euclidean distance metric (see Materials and Methods for details). The analysis time window of the longest vocalization was used (Meow 4) and the bin size of the PSTHs generated for each trial and neuron was varied. The percentage of times that the example neurons (presented in Figs. 1 and 2) were able to correctly discriminate these 15 different vocalizations is shown in Fig. 11A. The % correct classification by the neural discriminator was plotted as a function of the bin size. Since we presented 15 different vocalizations, the chance performance of the discriminator was 6.7% (dotted line). For both example neurons, the performance of the discriminator increased as the temporal resolution of the data became finer (decrease in bin size), confirming the above results that the different stimuli were better discriminated by the temporal pattern of the response than the mean firing rate. The % correct classification by PAF example neuron (triangles in Fig. 11A) was better than that of the AAF example (circles in Fig. 11A), consistent with the visual inspection that PAF neurons showed more variability in response pattern than AAF neurons (Figs. 1 and 2).

The above two examples were neurons that yielded the best discrimination performance in our AAF and PAF samples, respectively. The mean and SD of the % correct of 92 single AAF neurons and 102 single PAF neurons are presented in Fig. 11B. A tendency for the discrimination performance to increase with the decrease of bin size was also observed, but the maximum of mean % correct was only about 25% in both AAF and PAF neurons. There was no significant difference between the means of % correct of AAF and PAF neurons at any bin size (p>0.05, t-test).

**Figure 12:**
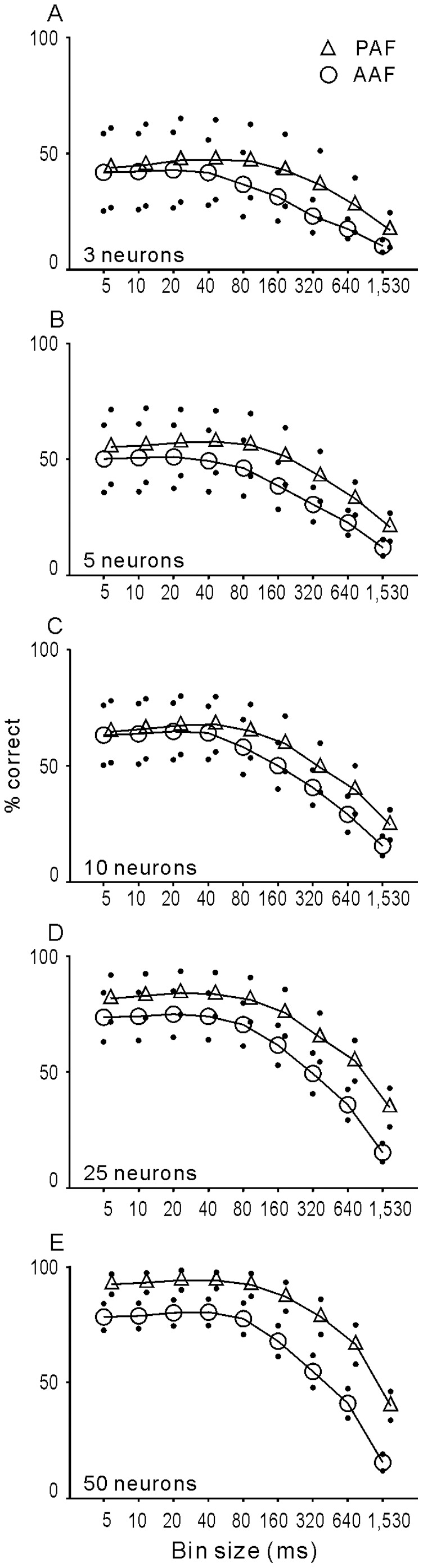
Accuracy of vocalization discrimination based on the spike activities of an ensemble of neurons in AAF and PAF. The results of 3, 5, 10, 25 and 50 randomly selected neurons are plotted as a function of the bin size in different panels. Circles (AAF) and triangles (PAF) are the mean values of 50 neuron selections and the dots represent the SD. To facilitate comparison, the functions of PAF are shifted slightly to the right. Asterisk indicates that the difference of mean values between AAF and PAF neurons is statistically significant (p<0.05, t-test).

While the % correct of 25% is obviously higher than the chance level of 6.7%, the discrimination of single neurons was inaccurate. We then examined whether the combined responses of multiple neurons can improve discrimination accuracy. For this, 3, 5, 10, 25 and 50 single neurons were randomly selected from AAF and PAF populations, respectively, and the neural discriminability computed based on the pooled spike activities. This computation was repeated 50 times with subsets drawn randomly from each cortical area every time. The mean % correct of these 50 repeats was taken as the representative value for a cortical area. The results of various numbers of neurons, shown as a function of % correct against the bin size, are presented in Fig. 12. It is clear that the % correct increased as the number of neurons increased and as the bin size decreased. When we included the spike activities of 50 neurons, the % correct reached 80% and 90% in AAF and PAF, respectively, suggesting that the temporal patterns of population responses carry sufficient information to allow for correct discrimination of stimuli that evoked these responses. More importantly, the performance of PAF neurons became significantly higher than that of AAF neurons (p<0.05, t-test), if the spike activities of 10 neurons were read out together in ≥80 ms bin size (Fig. 12C). Such a difference in % correct was observed for all bin sizes when the activities of 25 and 50 neurons were pooled (Fig. 12D and 12E). Therefore, although the discrimination accuracy of individual neurons was similar between AAF and PAF, the accuracy of neuron populations of PAF surpassed that of AAF. This may have been because there was more variability in the response patterns among PAF neurons than AAF neurons.

## Discussion

### Limits of the Study

In the present study, for the first time, we compared the properties of neural responses to vocalizations in AAF and PAF of awake cats. Preliminary to the general discussion, the limits of the present study on awake cats should be emphasized. Firstly, although the cat’s head was immobilized during the field-free experiments, we did not monitor pinna positions or the attention state and theoretically these may have contributed to response variability in this study [33]. Another potential source of response variation in this study is the likelihood that there was some variation in the recording layer. Although we did not assess the recording layers here, it is known that response properties change as a function of the cortical layer within A1 [34]. Combining neurons from multiple cortical layers may have increased the variability of neural responses in our dataset. Thirdly, because of the limited time for performing a single-unit recording in awake animals, we only used a small stimulus set to test the neurons. It could therefore not be excluded that the neurons may prefer some specific meows that were not tested in this study, such as hiss, purr, and growl. Nevertheless, in the current study, we focused on the comparison of neural response properties of AAF and PAF under the same experimental conditions.

### Differences in the Neural Responses of AAF and PAF

Our data indicated that PAF neurons had more heterogeneity of response patterns than AAF neurons. Most AAF neurons showed a transient excitatory response at stimulus onset/offset, and sometimes a suppressive response during the stimulus period. Such a response pattern was also found in some PAF neurons, but a substantial number of PAF neurons showed a sustained excitatory response during the stimulus period (Figs. 3, 4 and 5). Consequently, the mean duration of the excitatory response was longer in PAF neurons (Fig. 6A), but the mean duration of the suppressive response was longer in AAF neurons (Fig. 6B). Also, PAF neurons had a higher mean response magnitude (Fig. 6C), while the AAF neurons had a higher peak response magnitude (Fig. 6D).

The response pattern of AAF neurons is in agreement with previous studies on the A1 of anesthetized cats, which showed that cat vocalizations excited neurons in A1 largely at the onset and offset of the stimulus and caused inhibition or no response at all during the other parts of the sound [2], [7], [35]. Our previous study on the A1 of awake cats showed that some A1 neurons showed a sustained excitatory response during the stimulus period, and that these neurons were more frequently found in the region of A1 tuned to low frequencies [6]. In this study, sustained neural responses were also found in the low frequency area of PAF (Fig. 4), which was adjacent to the low frequency side of A1; therefore, the sustained response neurons were concentrated on the caudal division of the cat’s auditory cortex. The results of Gourevitch and Eggermont on anesthetized cats also showed a similar tendency [2]. The appearance of sustained firing suggests that the temporal integration window of the neurons to process sound stimuli is elongated. A recent study on marmosets found that more neurons used a rate code as the recording sites moved from A1 to the rostral field (R) of the core area, suggesting that the temporal integration window increases along the caudal-to-rostral axis of a monkey’s auditory cortex [36]. The monkey’s A1 and R are delineated by a reversal of frequency representation, and the border region between them represents low frequencies. The cat’s A1 and PAF have a similar relationship; hence, the PAF of a cat may correspond to the R of a monkey, and the temporal integration window increases along the rostral-to-caudal axis of a cat’s auditory cortex.

### Selectivity for Vocalizations

In this study, we found that most AAF and PAF neurons had low selectivity for our exemplars of vocalizations (Fig. 8), and there was no significant difference in responsiveness to the three stimulus categories: forward, reversed meows and human vowels (Fig. 9). Our previous study found a similar result on the A1 neurons of awake cats. Because both AAF and PAF have a tonotopic organization, mirroring that of A1 on the rostral and caudal sides, respectively [22]–[26], the three cortical fields of cats may be equivalent to the ‘auditory core’ of the monkey cortex [37]. By comparing the magnitude of vocalization-evoked response with the neuron’s BF, we found that stronger responses were concentrated in the area of BF <5 kHz in both AAF and PAF (Fig. 7), which matched the energy distribution of the vocalizations. Hence, AAF and PAF may still play a significant role in the representation of the physical parameters of sound stimuli.

Neural responses to forward and reversed conspecific vocalizations have been compared in previous studies on both cats [2], [7] and monkeys [5], [19], [38], and these studies generally concluded that the global firing rate in A1 and its adjacent areas was not largely modified by reversing the vocalization. Our data are consistent with these previous results. It should be noted that the spectro-temporal structure of meows is one that looks and sounds quite similar when reversed. To date, no behavioral experiments have examined whether cats perceive natural and reversed meows as different sound categories. Also, human vowels share a similar harmonic stack structure with very slow temporal dynamics; therefore, the similarity between neural responses may be due to the spectro-temporal similarity of these stimuli. One question remains open: is there any selectivity preference for meows over other dissimilar sounds, such as dog barks, human consonants and modulated noise?

On the other hand, neural responses preferring conspecific vocalizations were found in the higher stages of the auditory hierarchy. Such evidence has been well presented by studies on songbirds, whose auditory system shows clear anatomical parallels to the mammalian auditory system. For example, neurons in intermediate auditory processing stages (field L and cHV) had stronger responses to a conspecific song than to synthetic sounds that were designed to match the overall power spectra and AM spectra of songs [39]. Furthermore, in the specialized song system nuclei, auditory neurons show an extremely selective response for the bird’s own song, but a weak response to almost any other sounds, including conspecific songs [40], [41]. In non-human primates, neurons sensitive to conspecific vocalizations were reported in the lateral belt area of the auditory cortex and prefrontal cortical area [16]–[18], [42]–[44]. In cats, Gourevitch and Eggermont’s experiment [2] suggested that the indication of conspecific vocalizations may be detected in the posterior ectosylvian gyrus (EP) of a cat’s cortex; however, their data were obtained in anesthized cats and the sampling size was small (only 21 sites). Because conspecific vocalizations are intrinsically significant, it is suspected that their processing might differ between awake and unconscious animals, and the roles of EP and other high-order cortical areas in the processing of a cat’s meow are worthy of further examination in awake cats. Whether some areas of a cat’s cortex have a high preference for conspecific meows, corresponding to the belt or parabelt area in monkeys, remains an interesting question to be resolved in the future. To prove the neural specificity of meows, synthetic sounds matching different aspects of the spectral and temporal structure of the natural vocalizations should also be applied as a control in a future study.

### Neural Discrimination of Natural Sounds

Although neural selectivity based on a simple criterion (firing rate is beyond a threshold or not) was poor in most AAF and PAF neurons, the temporal pattern of neural responses can be used to discriminate vocalizations. Both information theory analysis and a linear pattern discriminator indicated that the accuracy of vocalization discrimination was better when fine temporal information was used than when only rate information was contained in the data sets (Figs. 10 and 11). Because PAF neurons showed more complex temporal response patterns, the maximum information (H_max_) carried by individual PAF neurons was generally higher than that carried by AAF neurons (Fig. 10C and 10D); however, the discrimination performance calculated by a linear pattern model was not significantly different between the AAF and PAF populations. The mean of % correct was only about 25% at the best temporal resolution in both cortical fields (Fig. 11B). A method that compensates for the imprecision of single-neuron encoding is to combine the activity of groups of neurons [45], [46]. Pooling the responses of multiple neurons in our sample obviously increased the performance of neural discrimination in both AAF and PAF, and the performance of PAF neuron groups was significantly higher than that of AAF neuron groups (Fig. 12).

A similar analysis of neural discriminators was also conducted on data from the monkey cortex by Recanzone [19], as well as by Russ et al. [17]. They also reported that the performance of the neural discriminator increased with the decrease of bin size, but the performance of individual neurons reached the maximum value of about 80–90% at a very short bin size of 2 ms. Recently, Kusmierek et al. pointed out that this result was somewhat perplexing, because rare neurons had the ability to lock to stimulus modulations at a frequency of 500 Hz (2 ms interval), and the algorithm of neural discriminators might not have been implemented precisely in their studies [20]. In this study, we paid special attention to our MATLAB scripts of discriminator analysis. Our results showed that the discrimination performance became saturated when the bin size was shorter than 40 ms, suggesting that reading the spike time on <40 ms scale cannot provide further information to enhance the discrimination performance. This is consistent with the calculation of Kusmierek et al. Other authors using neural discriminator methods to study cortical responses to sounds also showed that the optimal bin size ranged from 5 to 50 ms in the auditory cortex [47]–[49]. Regardless of the optimal bin size, our results suggest that AAF and PAF may use the temporal response patterns of neuron populations to represent vocalizations.

### Hierarchical Auditory Processing in Cat Auditory Cortex

It has been widely accepted that the cortical process of visual information is divided into two streams: a ventral or ‘what’ processing stream and a dorsal or ‘where’ processing stream. Based on the concept of parallel processing streams in the visual system, Rauschecker et al. have proposed that the auditory cortex may also contain separate processing streams that are specialized for either object discrimination or spatial processing [12]–[15]. Specifically, monkey electrophysiological studies suggest that cortical areas rostral to A1 may be specialized for auditory-object processing [8], [18], [21] and areas caudal to A1 may be specialized for accurately determining the spatial location of a sound source [50], [51]. To date, whether hierarchical organization of auditory processing exists in other species remains unclear.

The results of anatomic studies have indicated that there may be a complex scheme of auditory processing between AAF and PAF in cats. On one hand, AAF and PAF receive different projections from the medial geniculate body (MGB). AAF receives strong projections from the rostral pole of MGB and lesser projections from the ventral division of MGB, while PAF receives dense projections from the posterior portion of the ventral MGB and smaller projections from the dorsal MGB [52]–[54]. This suggests that AAF and PAF may work in parallel in auditory processing. On the other hand, there are some corticocortical connections projecting from AAF to A1, then from A1 to PAF, and rare connections projecting in the reverse direction. PAF also receives some direct projections from AAF [54], [55]. The existence of such functional corticocortical connections was also suggested by reversible deactivation studies on cats, in which neuronal silencing of anterior auditory cortical areas decreased the response properties of neurons in adjacent posterior cortical areas [56]–[58]. These results suggest that there may be a serial link between AAF and PAF. Consequently, a complex system including both serial and parallel processing pathways may exist between AAF and PAF in cats; that is, auditory information is processed in parallel, in which information from the thalamus arrives simultaneously at AAF and PAF, and is then modulated by corticocortical connections between them. Our present electrophysiological results, that both AAF and PAF neurons were well elicited by vocalizations and showed low selectivity, are attributable to both cortical areas receiving direct inputs from MGB in parallel. Thus, they belong to low-level stages of vocalization processing. The longer peak response latency, complex response pattern and higher capacity of vocalization discrimination in PAF neurons may be due to corticocortical modulation from AAF to PAF.

Recently, Carrasco and Lomber systemically compared the latencies of neural responses to various sounds in AAF, A1, the secondary auditory field (A2, adjacent to A1 on the ventral side) and PAF of anesthetized cats, and found a substantial increase in response latency along the sequence of AAF, A1, A2 and PAF [28]. Based on the difference in response latencies among the auditory cortical fields, and the well-established system of visual processing, they proposed that “anterior auditory cortical fields are good candidates for the early analysis of low-level stages of acoustic processing, while posterior fields are good candidates for the latter analysis of high-level acoustic scenes”. Our present results are in general agreement with this proposal; however, a behavioral experiment showed that bilateral deactivation of the AAF resulted in deficits in a pattern-discrimination task, whereas bilateral deactivation of the PAF resulted in deficits in a sound-localization task [27]. This suggests that AAF may be more involved in the perception of an acoustic object than PAF. One point should be noted that an acoustic object can be defined in many different ways [59]. In the above-mentioned study, the cats were examined using a task to discriminate different gap sequences embedded in broadband noise bursts. This task only examined the perceptual attributes of the temporal pattern of the sound envelope. As shown in Fig. 3 in our data, most AAF neurons showed a transient response to the abrupt change of the sound envelope at stimulus onset and offset. With temporally precise responses, AAF neurons could present a robust representation of the gaps among noise bursts. In this regard, it is understandable that AAF deactivation caused a deficit in temporal pattern discrimination; however, cat vocalizations and human vowels contained less abrupt changes of the sound envelope, and spectral information, such as fundamental frequency and pitch, may play a more important role in vocalization discrimination. How the perception of spectral information is affected by the deactivation of AAF and PAF has not been examined yet; therefore, it is still premature to rule out PAF’s involvement in the processing of acoustic objects. Although we agree with the hypothesis that ‘what’ and ‘where’ streams may exist in the auditory cortex of cats, the two streams may not completely separate in the early auditory cortex of AAF and PAF. In the future, more attempts will be needed to find the cat cortical areas specialized in processing acoustic objects.

## Materials and Methods

All animal work was carried out in strict accordance with the recommendations in the Guide for the Care and Use of Laboratory Animals of the National Institutes of Health. The protocol was approved by the Committee on the Ethics of Animal Experiments of the University of Yamanashi (permit number No. 19-15). All surgery was performed under sodium pentobarbital anesthesia, and all efforts were made to minimize suffering.

### Surgical Preparation, Electrophysiological Recording, and Histology

Animal preparation and recording procedures were similar to those used in our previous experiments [6], [60], [61]. Under pentobarbital sodium anesthesia and aseptic conditions, an aluminum cylinder (inner diameter, 20 mm) was implanted bilaterally into the temporal bone for microelectrode access. A metal block was embedded in a dental acrylic cap to immobilize the head. After 2–3 weeks of postoperative recovery and adaptation training, recording experiments were performed in an electrically shielded, sound-attenuated chamber. A 0.5 mm diameter hole was drilled in the skull, the dura was pierced with a sharpened probe, and then a single epoxylite-insulated tungsten microelectrode (FHC Inc.; impedance: 2–5 M*Ω* at 1 kHz) was advanced into the auditory cortex using a remote-controlled micromanipulator (MO-951; Narishige). The coordinate of each electrode site was read from the MO-951 scales and calibrated to a fixed mark inside the recording chamber. Well-isolated single units were discriminated using a template-matching discriminator (ASD; Alpha-Omega Engineering) in 50 µs time resolution. The digital ASD outputs of the spike occurrence time (time resolution: 50 µs) were stored on a hard disk.

Daily recording sessions lasted 3–5 hours over 2–6 months. During the recording period, the cat’s head was immobilized in a custom-built frame through a metal block, and the body was wrapped in a cotton bag. The cats had been pre-trained to become accustomed to this condition. A video camera was placed in front of the cat to monitor its state. No sign of discomfort was observed as the cats passively listened to the auditory stimuli.

At the end of the experiment, several recording sites were re-approached and marked by electrolytic lesions. The animal was then deeply anesthetized with sodium pentobarbital and perfused with 10% formalin before the brain was removed. The brain surface was photographed. The cerebral cortex was cut in coronal sections and stained with neutral red. The location map of recording sites was constructed on the brain surface by calibrating the coordinates of the lesion sites.

### Acoustic Stimulus Preparation and Presentation

Acoustic stimuli were presented from a speaker placed 2 cm from the auricle contralateral to the recording site. The sound-delivering system was calibrated to produce a flat spectrum (128–32,000 Hz, ±5 dB) measured at the entrance of the cat’s meatus. For each isolated unit, we applied a set of pure-tone bursts (160 ms in duration, including 5 ms rise/fall time) to access the tuning property, which was used to identify the recording location. Then, a set of communication sounds was presented. This stimulus set was identical to that used in our previous study [6], including five cat vocalizations (meow calls) presented in forward and time-reversed directions and five human vowels (/a, o, u, e, i/) in Japanese. The spectrographic representations of the stimuli are illustrated in Fig. 1. Meow calls were collected from spontaneously vocalizing cats recorded individually in a sound attenuated room. The sounds were recorded by a microphone (LA-5110; ONO SOKKI). The recorded signals were passed through a low-pass filter (cutoff frequency = 20 kHz), then connected to a computer and digitized using Spike2 software (Cambridge Electronic Design) with a sampling rate of 100 kHz. The vowels were recorded under the same conditions by a male Japanese speaker. The forward, time-reversed meows, and vowels were presented in random order at a peak level of 50 dB sound pressure level (SPL, dB re 20 µPa). Each vocalization was repeated 8 times (inter-stimulus interval >1.5 s).

### Data Analysis

As shown in Fig. 1, the spike trains of each neuron were aligned along the onset of stimulus to construct a raster plot of each stimulus condition. The peri-stimulus time histogram (PSTH) of the firing rate was computed in 1-ms bin width across 8 trials of the same stimulus and convolved with a Gaussian kernel (σ = 10). The spontaneous firing rate for 500 ms before stimulus onset was considered as background. The mean +2SD of background firing rates across the trials of all stimuli was deemed as the threshold level to identify a significant response. The analysis time window to access a stimulus-evoked response was set from stimulus onset to 50 ms after stimulus offset, in order to include both onset and offset responses. During this time window, a firing rate that was higher than the threshold level for 10 consecutive 1 ms bins was designated an evoked excitatory response, whereas firing rate < mean –2 SD was designated a suppressive response. We defined a neuron as “vocalization responsive” if at least one of the vocalization stimuli elicited an excitatory or suppressive response that met the above criteria.

To illustrate the response patterns of the neuron population, we constructed a color-coded spike density histogram for each neuron (Fig. 3). For visualization purposes, the firing rate of each neuron was assessed by the normalized value of the Z-score [62], which was calculated by subtracting the mean background firing rate (averaged across the all trials of 500 ms pre-stimulus period) from the firing rate, and then divided by the SD of the background firing rate. White in the plot indicates lower than the background firing rate. Black indicates firing that is ≥4 SD of the background firing rate.

Duration of the excitatory/suppressive response was the summation of time bins, at which the criterion of excitatory or suppressive response was met, during the analysis time window. Peak latency for each neuron was defined as the time at which the spike density function reached its maximum. We also calculated both the mean and peak response magnitudes. Mean response magnitude was defined as the mean firing rate during the analysis time window. Peak magnitude was defined as the average firing rate for 25 ms on either side of the peak latency. Both response magnitudes were defined as firing rates above the baseline activity of the neurons (driven rate).

### Information Theory Analyses of Temporal Coding in Responses from Single Neurons

The information content of neural responses was analyzed as previously described [63], using metric space analyses (for further details, see [64], [65]). This analysis quantified the amount of information about vocalization identity contributed by spike count alone and the precise timing of spikes. The key quantity is the “distance” between two spike trains, which is quantified as the minimum cost of inserting, deleting, or moving spikes in one train, to make one spike train match the other. The cost of inserting or deleting a spike from a spike train is always set at 1. The cost of moving a spike (per unit time) is q, which is varied parametrically to examine responses at multiple levels of temporal precision. The distance between two spike trains in terms of spike count alone is the cost obtained when q = 0 (because there is no cost associated with moving a spike); this is simply the difference between the spike count of each response and is called D^count^. To take into account the temporal characteristics of the spike trains, we calculate the distance for a range of values q>0, denoted D^spike^[q]. The parameter q (in units of 1/second) determines the temporal precision of the analysis, since the cost of moving a spike by an amount 1/q is equal to the cost of deleting it altogether. Calculations were performed for q = 0, and in octave steps from q = 5/ms to 640/ms. Pairwise distances between all responses are computed for a given value of q and clustering is performed.

Clusters can be defined as sets of spike trains that are close to each other. To evaluate the extent of the similarity between spike trains elicited by the same stimulus and those elicited by another stimulus, a confusion matrix N(s_i_,r_j_) is constructed. This matrix summarizes, for each stimulus class, how many spike trains can be attributed to this class, based on the average distance (or similarity) of this spike train from other spike trains of the same stimulus class. This matrix N is then used to compute the amount of transmitted information H:
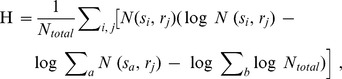



in which *N*_total_ is the total number of spike trains and logarithms are in base 2. The value of the information, H, indicates the performance of stimulus-dependent clustering based on the temporal patterns of neural responses (i.e., how well the responses can be decoded). Animals in our experiment were presented with 15 stimuli; the amount of information required for perfect discrimination among 15 stimuli is 3.9 bits. If the clustering is totally random, H = 0.

The value of information for q = 0 is denoted as H_0_ and indicates information conveyed by spike count alone (i.e., a rate code). The value of q at which H is greatest is referred to as q_max_ and the maximal value of H (i.e., its value at q_max_) is H_max_. When H_max_ was greater than the value of H at q = 0 (H_count_), the time course of the response and/or the timing of individual spikes contributed information above that contained in the spike count.

Amounts of information computed from experimental data are biased estimates of the “true” transmitted information that would ideally be obtained from an infinite number of trials for each stimulus; therefore, a control computation was performed to estimate the bias (or chance level) and to assess the significance of transmitted information resulting from the calculation described above. Metric space analysis was repeated for data sets in which the stimuli associated with the spike trains were randomly shuffled. In reporting the results, only values of H that were at least 2 SDs greater than the average amount of information in the shuffled datasets (H_shuffled_) were considered significant and therefore those neurons were denoted informative neurons. The above calculations were performed using the Spike Train Analysis Toolkit (http://neuroanalysis.org/toolkit/) and in-house MATLAB software.

### Linear Pattern Discriminator

A linear pattern discriminator based on the spike distance metric (SDM) was used to test how well the responses of a neuron differentiate different stimuli [17], [19], [29], [48], [66]–[69]. For each neuron, a PSTH of one trial was chosen and removed from the data, referred to as the test trial. The remaining trials were grouped into 15 sets according to the actually presented stimulus (7 trials for the selected stimulus, 8 trials for the other 14 stimuli). A template PSTH of each stimulus was then constructed using the mean of each set of remaining trials.

Next, we examined whether we could determine which stimulus elicited the test PSTH by comparing how similar the test PSTH was to the 15 PSTH templates. For this, we calculated the Euclidian distance (ED) between the test and template PSTH, which is the square root of the sum of squared differences between firing rates at each bin (i).

ED = 
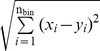
, where n_bin_ is the total number of bins, and x and y are bin heights.

If the test and template PSTH were similar, then ED would be small. If they were different, then ED would be large. The test PSTH was assigned to be the stimulus that had the most similar training set. This procedure was repeated until each trial of a neuron was considered as test data. Percentage correct for each neuron was calculated as the percentage of total number of trials in which the correct stimulus was selected.

This analysis was conducted during 0–1,530 ms after stimulus onset, covering the duration of our longest stimulus (Meow 4) and 50 ms post-stimulus period. PSTH during this period was constructed in various bin sizes to explore the temporal precision of neural encoding. A larger bin size will capture more firing rate information, while a smaller bin size will preserve more spike time information. All neurons within a given cortical area were tested using bin sizes of 5, 10, 20, 40, 80, 160, 640 and 1,530 ms. The 5 ms bin size retains the most temporal information, whereas the 1,530 ms bin is equivalent to the mean response magnitude.
